# Genomewide Assessment of Mycobacterium tuberculosis Conditionally Essential Metabolic Pathways

**DOI:** 10.1128/mSystems.00070-19

**Published:** 2019-06-25

**Authors:** Yusuke Minato, Daryl M. Gohl, Joshua M. Thiede, Jeremy M. Chacón, William R. Harcombe, Fumito Maruyama, Anthony D. Baughn

**Affiliations:** a Department of Microbiology and Immunology, University of Minnesota Medical School, Minneapolis, Minnesota, USA; b University of Minnesota Genomics Center, Minneapolis, Minnesota, USA; c Biotechnology Institute and Department of Ecology, Evolution and Behavior, University of Minnesota, St. Paul, Minnesota, USA; d Department of Microbiology, Graduate School of Medicine, Kyoto University, Kyoto, Japan; e The Japan Science and Technology Agency/Japan International Cooperation Agency, Science and Technology Research Partnership for Sustainable Development (JST/JICA, SATREPS), Tokyo, Japan; f Scientific and Technological Bioresource Nucleus, Universidad de La Frontera, Temuco, Chile; University of California, San Diego

**Keywords:** comparative genomics, metabolic modeling, metabolism, Tn-seq, tuberculosis

## Abstract

A better understanding of essential cellular functions in pathogenic bacteria is important for the development of more effective antimicrobial agents. We performed a comprehensive identification of essential genes in Mycobacterium tuberculosis, the major causative agent of tuberculosis, using a combination of transposon insertion sequencing (Tn-seq) and comparative genomic analysis. To identify conditionally essential genes by Tn-seq, we used media with different nutrient compositions. Although many conditional gene essentialities were affected by the presence of relevant nutrient sources, we also found that the essentiality of genes in a subset of metabolic pathways was unaffected by metabolite availability. Comparative genomic analysis revealed that not all essential genes identified by Tn-seq were fully conserved within the M. tuberculosis complex, including some existing antitubercular drug target genes. In addition, we utilized an available M. tuberculosis genome-scale metabolic model, iSM810, to predict M. tuberculosis gene essentiality *in silico*. Comparing the sets of essential genes experimentally identified by Tn-seq to those predicted *in silico* reveals the capabilities and limitations of gene essentiality predictions, highlighting the complexity of M. tuberculosis essential metabolic functions. This study provides a promising platform to study essential cellular functions in M. tuberculosis.

**IMPORTANCE** Mycobacterium tuberculosis causes 10 million cases of tuberculosis (TB), resulting in over 1 million deaths each year. TB therapy is challenging because it requires a minimum of 6 months of treatment with multiple drugs. Protracted treatment times and the emergent spread of drug-resistant M. tuberculosis necessitate the identification of novel targets for drug discovery to curb this global health threat. Essential functions, defined as those indispensable for growth and/or survival, are potential targets for new antimicrobial drugs. In this study, we aimed to define gene essentialities of M. tuberculosis on a genomewide scale to comprehensively identify potential targets for drug discovery. We utilized a combination of experimental (functional genomics) and *in silico* approaches (comparative genomics and flux balance analysis). Our functional genomics approach identified sets of genes whose essentiality was affected by nutrient availability. Comparative genomics revealed that not all essential genes were fully conserved within the M. tuberculosis complex. Comparing sets of essential genes identified by functional genomics to those predicted by flux balance analysis highlighted gaps in current knowledge regarding M. tuberculosis metabolic capabilities. Thus, our study identifies numerous potential antitubercular drug targets and provides a comprehensive picture of the complexity of M. tuberculosis essential cellular functions.

## Abstract (precis)

Mycobacterium tuberculosis causes 10 million cases of tuberculosis (TB), resulting in over 1 million deaths each year. TB therapy is challenging because it requires a minimum of 6 months of treatment with multiple drugs. Protracted treatment times and the emergent spread of drug-resistant M. tuberculosis necessitate the identification of novel targets for drug discovery to curb this global health threat. Essential functions, defined as those indispensable for growth and/or survival, are potential targets for new antimicrobial drugs. In this study, we aimed to define gene essentialities of M. tuberculosis on a genomewide scale to comprehensively identify potential targets for drug discovery. We utilized a combination of experimental (functional genomics) and *in silico* approaches (comparative genomics and flux balance analysis). Our functional genomics approach identified sets of genes whose essentiality was affected by nutrient availability. Comparative genomics revealed that not all essential genes were fully conserved within the M. tuberculosis complex. Comparing sets of essential genes identified by functional genomics to those predicted by flux balance analysis highlighted gaps in current knowledge regarding M. tuberculosis metabolic capabilities. Thus, our study identifies numerous potential antitubercular drug targets and provides a comprehensive picture of the complexity of M. tuberculosis essential cellular functions.

## INTRODUCTION

Mycobacterium tuberculosis is responsible for approximately 10.4 million new cases of active tuberculosis (TB) infection and 1.4 million deaths annually (1). While TB chemotherapy has a high success rate in curing drug-susceptible TB infections, it is challenging, in part because it requires a minimum of 6 months of treatment with drugs associated with adverse reactions. Thus, finding targets for new TB drugs that are more potent than existing drugs is needed (2).

Essential genes, defined as genes indispensable for growth and/or survival, are potential targets for new types of antimicrobial drugs. Gene essentiality can be assessed by targeted gene disruptions, where genes that cannot be disrupted are typically categorized as being essential. However, such traditional genetic approaches are labor-intensive and not easily adaptable to genome-scale screening. Recent advances in next-generation sequencing (NGS)-based approaches have transformed our ability to examine gene functions in a genomewide manner. Transposon insertion sequencing (Tn-seq) has been widely used to conduct fitness profiling of gene functions in many bacterial species, including M. tuberculosis (3–10). In addition to fitness profiling, a lack of representation of specific transposon insertions within a saturated transposon library has been used to identify essential genes in genomewide screens (3, 9, 10).

In M. tuberculosis, several systematic genomewide studies, including studies using Tn-seq, have been performed to identify essential genes *in vitro* (7–16) and *in vivo* (4). The gene essentialities determined by Tn-seq studies are accessible through publicly available databases, such as TubercuList (17), BioCyc (18), and the *o*nline *ge*ne *e*ssentiality (OGEE) database (19). Most of these gene essentiality data were obtained from Tn-seq studies that were carried out using a defined growth medium that was supplemented with a limited number of nutrients (7–10, 13, 15). Under this growth condition, genes for numerous essential central metabolic pathways can be rendered dispensable through supplementation. Thus, these previous studies have likely miscategorized a large set of genes as essential rather than conditionally essential. For example, pantothenate, an essential precursor in coenzyme A biosynthesis, is not supplemented in most of the commonly used media for M. tuberculosis. As a result, *panC*, encoding pantothenate synthetase, the enzyme catalyzing the last step in pantothenate biosynthesis, was defined as an essential gene in these three databases (17–19) despite a previous study showing that the *panCD* double deletion mutant of M. tuberculosis H37Rv strain can grow in the presence of supplemental pantothenate (20). Thus, more precise definition of essential M. tuberculosis genes on a genomewide scale and annotation of genes which are conditionally essential would improve the usefulness of these databases.

In this study, we developed a defined-nutrient-rich medium for M. tuberculosis (MtbYM rich medium, where YM stands for yareplete metabolite) that included a variety of nutrient sources for M. tuberculosis to ease identification of conditionally essential genes. We used Tn-seq to identify essential genes in MtbYM rich and minimal (Mtbminimal) media. As expected, the essentialities of many genes involved in metabolite biosynthesis pathways were affected by the supplemental nutrients in MtbYM rich medium. However, we found that essentialities of certain metabolic pathways were unaffected by the absence or presence of relevant nutrient sources. We also found that some essential genes were unique to each growth condition. In addition, we compared essential genes identified by Tn-seq with highly conserved genes that were identified by a comparative genomics analysis and a modified *in silico* metabolic model. These comparisons indicated that essential genes were highly enriched among the conserved core genome and that such gene essentiality measurements can be used to refine metabolic models.

## RESULTS AND DISCUSSION

### Identification of essential M. tuberculosis genes in a chemically defined nutrient-rich medium.

**FIG 1:**
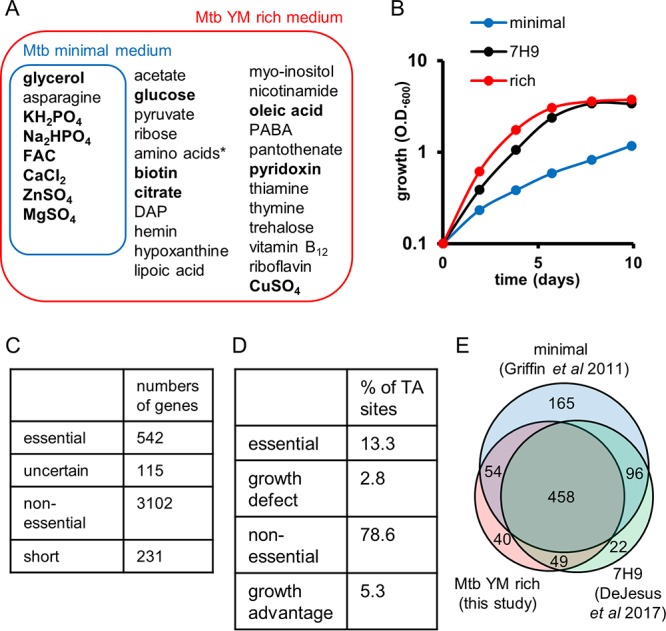
Identification of essential genes in MtbYM rich medium. (A) Medium compositions of Mtbminimal and MtbYM rich media. 7H9 medium contains glutamic acid and ammonium sulfate in addition to the nutrients shown in bold. DAP, diaminopimelic acid; FAC, ferric ammonium citrate; PABA, *para*-aminobenzoic acid; *, 0.5% Casamino Acids and 0.98 mM tryptophan. (B) M. tuberculosis H37Rv growth in MtbYM rich (red), 7H9 (black), and Mtbminimal (blue) media. (C) Bayesian/Gumbel results for M. tuberculosis H37Rv in MtbYM rich medium. (D) HMM results for M. tuberculosis H37Rv in MtbYM rich medium. (E) Venn diagram comparisons of essential genes by Tn-seq in this study and two previous studies (8, 9). Red, this study; blue, Griffin et al. (8); green, DeJesus et al. (9).

We developed a defined-nutrient-rich medium for M. tuberculosis (MtbYM rich medium) (Fig. 1A; see also Table S1 in the supplemental material) by supplementing numerous nutrients into a minimal M. tuberculosis growth medium (Mtbminimal medium) (21). We chose supplements based on their known use by various bacterial species, including M. tuberculosis (7, 12, 20, 22–24). The supplements included several carbon sources, nitrogen sources, cofactors, amino acids, nucleotide bases, and other nutrients. Four of the supplements, lipoic acid, nicotinamide, hemin, and ribose, inhibited M. tuberculosis H37Rv growth at high concentrations. Thus, we utilized concentrations of these supplements that did not impair growth. The MtbYM rich medium supported M. tuberculosis H37Rv’s growth similarly to the commonly used 7H9 medium, which contains essential salts and relatively few nutrients (Fig. 1B).

10.1128/mSystems.00070-19.2 TABLE S1 Compositions of MtbYM rich and Mtbminimal media. Download Table S1, XLSX file, 0.01 MB. Copyright © 2019 Minato et al. 2019 Minato et al. This content is distributed under the terms of the Creative Commons Attribution 4.0 International license.

We next generated a library of M. tuberculosis H37Rv transposon insertion mutants on MtbYM rich medium plates. As the M. tuberculosis H37Rv genome contains 74,602 TA dinucleotides, we collected at least 150,000 colonies to approach saturation of *himar1* transposon insertion sites in the library. Genomic DNA (gDNA) was isolated from the library. Next, the DNA was sheared and end repaired, sequencing adapters were added, and the transposon adjacent regions were enriched by PCR before massively parallel sequencing. The resultant sequencing data were analyzed using TRANSIT, a recently developed software package for analyzing Tn-seq data (14). TRANSIT contains two statistical methods, the Bayesian/Gumbel method and the hidden Markov model (HMM), to identify essential genes and essential genomic regions, respectively, under a single growth condition. By using the Bayesian/Gumbel method, we found that 542 genes were essential for M. tuberculosis growth in the MtbYM rich medium (Fig. 1C; Data Set S1). The Bayesian/Gumbel method assesses gene essentiality based on consecutive sequences of TA sites lacking insertion within a gene. When the analysis did not exceed the significance thresholds, genes were called either short or uncertain by TRANSIT. As a result, 231 genes were called short and 115 genes were called uncertain by this analysis (Fig. 1C). To overcome this issue, we also identified essential genomic regions using an HMM (14). The HMM is based on the read count at a given site and the distribution over the surrounding sites. This analysis identified 13.3% of TA sites as essential and 2.8% of TA sites as growth defective (Fig. 1D; Data Set S1). These essential and growth-defective regions included 17 short genes (14 essential and 3 growth-defective genes) and 21 uncertain genes (10 essential and 11 growth-defective genes) whose essentiality could not be assessed by the Bayesian/Gumbel method. In addition, HMM identified 5 short genes and 16 uncertain genes that contained both essential and nonessential TA sites. These genes are listed in Table S2. In total, we identified 601 genes (542 genes by the Bayesian/Gumbel method and an additional 59 genes by the HMM) as essential genes for M. tuberculosis survival in the MtbYM rich medium. A list of these genes is available through the BioCyc smart table format (https://biocyc.org/group?id=biocyc14-7907-3764257976) (25). These essential genes included known targets for existing antitubercular drugs, further validating that the Tn-seq assay successfully identified essential M. tuberculosis genes (Table 1).

**TABLE 1:** Gene essentialities and *in silico* essentiality predictions of antitubercular drug target genes^*a*^

Gene (locus tag)	Drug(s)	Presence of core/soft core gene (% of strains in which it is conserved)
*inhA* (*Rv1484*)	Isoniazid-ethionamide	Yes (96)
*embA* (*Rv3794*)	Ethambutol	No (79)
*embB* (*Rv3795*)	Ethambutol	No (83)
*rpoB* (*Rv0667*)	Rifampin	Yes (97)
*atpE* (*Rv1305*)	Bedaquiline	Yes (100)
*gyrA* (*Rv0006*)	Fluoroquinolones	Yes (99)
*gyrB* (*Rv0005*)	Fluoroquinolones	No (89)
*dfrA* (*Rv2763c*)	*para*-Aminosalicylic acid	Yes (99)
*alr* (*Rv3423c*)	d-Cycloserine	Yes (98)

a Every listed gene was essential in MtbYM rich medium.

10.1128/mSystems.00070-19.3 TABLE S2 List of short and uncertain genes that were identified as essential by the TRANSIT HMM. Download Table S2, XLSX file, 0.01 MB. Copyright © 2019 Minato et al. 2019 Minato et al. This content is distributed under the terms of the Creative Commons Attribution 4.0 International license.

10.1128/mSystems.00070-19.9 DATA SET S1 Analysis of Tn-seq gene essentiality analysis by the TRANSIT Bayesian/Gumbel method and hidden Markov model. Download Data Set S1, XLSX file, 4.2 MB. Copyright © 2019 Minato et al. 2019 Minato et al. This content is distributed under the terms of the Creative Commons Attribution 4.0 International license.

**FIG 2:**
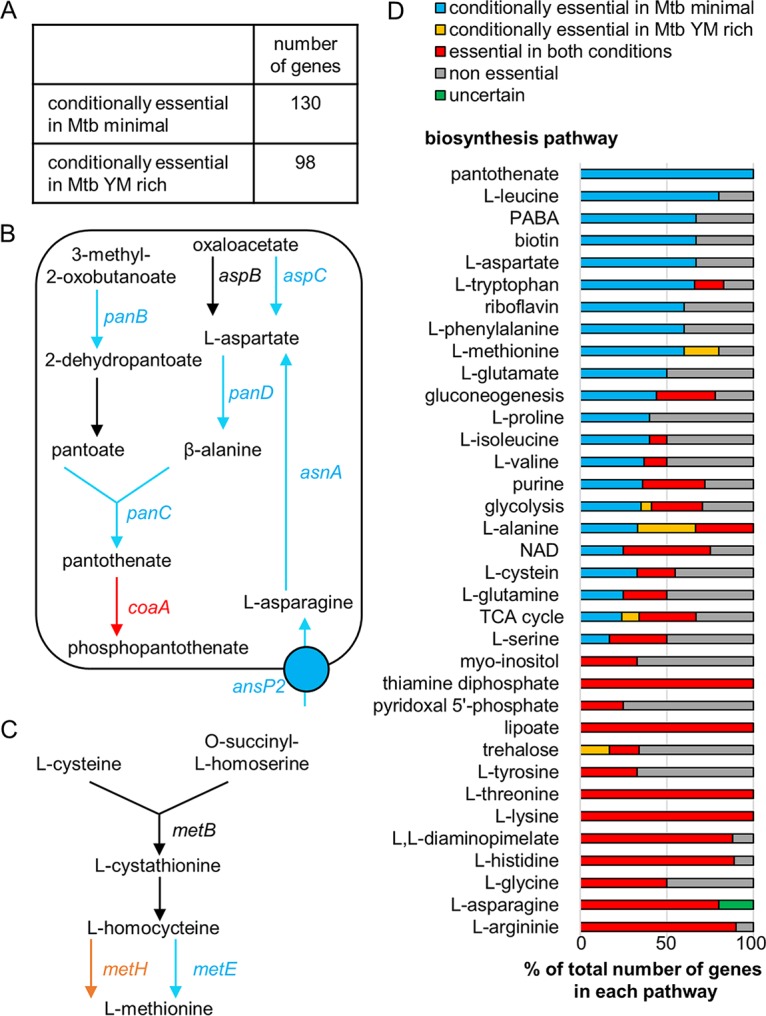
Identification of conditionally essential genes in MtbYM rich and Mtbminimal media. (A) Results for comparative analysis between MtbYM rich and Mtbminimal media. Numbers of conditionally essential genes (adjusted *P* value of <0.05) were identified by the resampling method in TRANSIT (14). (B, C) Essentialities of genes in the pantothenate biosynthesis pathway (B) and methionine biosynthesis pathway (C). Genes in red are essential in both MtbYM rich and Mtbminimal media, genes in blue are essential in Mtbminimal medium but not essential in MtbYM rich medium, genes in orange are essential in MtbYM rich medium but not essential in Mtbminimal medium, and genes in black are nonessential. Information on genes in each pathway was obtained from the BioCyc database (18). (D) Ratios of essential genes in the metabolic pathways. All genes are listed in Table S5. PABA, *para*-aminobenzoic acid; purine, 5-aminoimidazole ribonucleotide.

The list of essential genes identified in this study was also compared with the essential genes identified by the past Tn-seq studies, Griffin et al. (8) and DeJesus et al. (9) (Fig. 1E and Table S3). In general, our result was largely consistent with those of the past studies, and a total of 458 genes were identified as essential in all three studies (Fig. 1E) (https://biocyc.org/group?id=biocyc14-7907-3764424447). Since MtbYM rich medium used in this study was supplemented with numerous nutrients, many genes that were related to biosynthetic pathways of the supplemented nutrients (e.g., amino acids, pantothenate, purine, flavin, and others) were not essential in our study, while the past studies categorized these genes as essential. We also identified genes that were essential only in our study and not in other studies. We identified some of these genes as conditionally essential in MtbYM rich medium (described below) (Fig. 2 and Table S4). Of note, our study could not detect several genes that were highly expected to be essential and were identified as essential in the DeJesus et al. study. These genes included short genes, such as several ribosomal genes and *folK*. This likely was because the DeJesus et al. study used a more saturated transposon library (14 replicates versus 2 replicates).

10.1128/mSystems.00070-19.4 TABLE S3 List of essential genes identified in this study and past studies. Download Table S3, XLSX file, 0.03 MB. Copyright © 2019 Minato et al. 2019 Minato et al. This content is distributed under the terms of the Creative Commons Attribution 4.0 International license.

10.1128/mSystems.00070-19.5 TABLE S4 Comparison of essential genes between MtbYM rich and Mtbminimal media identified by Tn-seq and analyzed by the TRANSIT resampling method. Download Table S4, XLSX file, 0.4 MB. Copyright © 2019 Minato et al. 2019 Minato et al. This content is distributed under the terms of the Creative Commons Attribution 4.0 International license.

10.1128/mSystems.00070-19.6 TABLE S5 Comparison of essential metabolic pathways between MtbYM rich and Mtbminimal media. Download Table S5, XLSX file, 0.01 MB. Copyright © 2019 Minato et al. 2019 Minato et al. This content is distributed under the terms of the Creative Commons Attribution 4.0 International license.

### Comparison of essential M. tuberculosis genes found with MtbYM rich medium and minimal-nutrient medium.

Because gene essentiality can be affected by the external environment (26), in particular by nutrient availability, we next compared gene essentialities found with MtbYM rich medium and Mtbminimal medium. As with the transposon library generated on MtbYM rich medium plates, we generated M. tuberculosis H37Rv transposon libraries on Mtbminimal medium plates and collected at least 150,000 colonies to create a saturated library. The resultant sequencing data were compared with the MtbYM rich plate data by using a permutation test-based method to identify genes with statistically significant differences in transposon insertion count (Fig. 2A; Table S4). We found that 130 genes were conditionally essential in the Mtbminimal medium compared to those found with the MtbYM rich medium (https://biocyc.org/group?id=biocyc13-7907-3706551227).

As anticipated, many of the conditionally essential genes that were identified corresponded to the differences in nutrient composition between Mtbminimal and MtbYM rich. For example, MtbYM rich medium contained l-aspartate and pantothenate. Thus, the genes in the l-aspartate and pantothenate biosynthesis pathways were dispensable in MtbYM rich medium (Fig. 2B). However, MtbYM rich medium did not contain metabolites located downstream of pantothenate. Consequently, these downstream genes (e.g., *coaA*, encoding pantothenate kinase) were essential in both MtbYM rich and Mtbminimal media (Fig. 2B). This observation is consistent with a previous report that the M. tuberculosis *panCD* double-deletion mutant strain can grow in the presence of pantothenate (20) and also suggested that single gene disruptions in the pantothenate biosynthesis pathway (*panB*, *panC*, and *panD*) show a pantothenate auxotrophic phenotype similar to that of the *panCD* double-deletion mutant strain. Another example was the asparagine transporter gene *ansP2* (27), which was conditionally essential in Mtbminimal medium (Fig. 2B), presumably because Mtbminimal medium contained arginine as the sole nitrogen source whereas MtbYM rich medium contained multiple nitrogen sources (Fig. 1A).

Unexpectedly, we also identified 98 genes that were conditionally essential in MtbYM rich medium compared to those found with the Mtbminimal medium (https://biocyc.org/group?id=biocyc13-7907-3710604059). Such genes included *ponA1* and *ponA2*, encoding penicillin binding proteins (PBPs) involved in cell wall peptidoglycan (PG) biogenesis. It was previously shown that *ponA1* and *ponA2* are essential only *in vivo*, not during growth in culture medium (28, 29). Thus, one of the nutrients that is uniquely present in MtbYM rich medium might also be present *in vivo* and may be responsible for the *in vivo* fitness defect of the *ponA1* mutant. LdtB is one of the major l,d-transpeptidases (Ldts) that is also involved in PG biogenesis. *ldtB* was also conditionally essential in MtbYM rich medium. Interestingly, several genes that were conditionally essential in the absence of *ponA1*, *ponA2*, or *ldtB* (e.g., *Rv1086*, *Rv1248c*, *Rv3490*, *treS*, and *otsA*) were also conditionally essential in MtbYM rich medium, suggesting that MtbYM rich medium negatively affects PG biogenesis in M. tuberculosis (30). We also found that *metH*, encoding one of the two methionine synthases, was essential only in MtbYM rich medium but that *metE*, encoding the other methionine synthase, was essential only in Mtbminimal medium (Fig. 2C). The observed conditional essentialities were consistent with those of a previous study showing that M. tuberculosis *metE* expression was inhibited in the presence of vitamin B_12_ by a *metE* B_12_ riboswitch and that vitamin B_12_-dependent MetH is used predominantly when vitamin B_12_ is available (31). These findings confirmed that the conditionally essential genes identified by Tn-seq in this study are consistent with previous findings (25).

The supplementation of nutrients in the MtbYM rich medium may subvert the need for enzymes in at least 35 metabolic pathways (Fig. 1A and 2D and Table S5). We found fewer gene essentialities in 22 of these pathways in MtbYM rich medium, suggesting that M. tuberculosis can functionally utilize these nutrients. Notably, many genes in these pathways were identified as essential genes by the previous studies (8, 13) and listed as essential genes in public databases, such as TubercuList (17) and BioCyc (18). Thus, our results revealed that these genes are essential only in the absence of the corresponding nutrients. In contrast, we also found that the essentiality of genes in 13 other metabolic pathways were not altered by nutrient supplementation (Fig. 2D). Previous studies have demonstrated that some auxotrophic mutants, such as those with mutations in l-arginine, l-lysine, and inositol, require supplementation with the corresponding nutrient at a relatively high concentration to support their growth (32–34). Certain auxotrophic mutants are also known to show growth or survival defects even in the presence of the corresponding nutrient (35, 36). These results were consistent with previous findings that the essentiality of some central metabolic pathways could not be bypassed in pathogenic mycobacteria (12) and also provide a more comprehensive understanding of the nutrient utilization capacity of M. tuberculosis.

### Identification of highly conserved genes in the M. tuberculosis complex.

**FIG 3:**
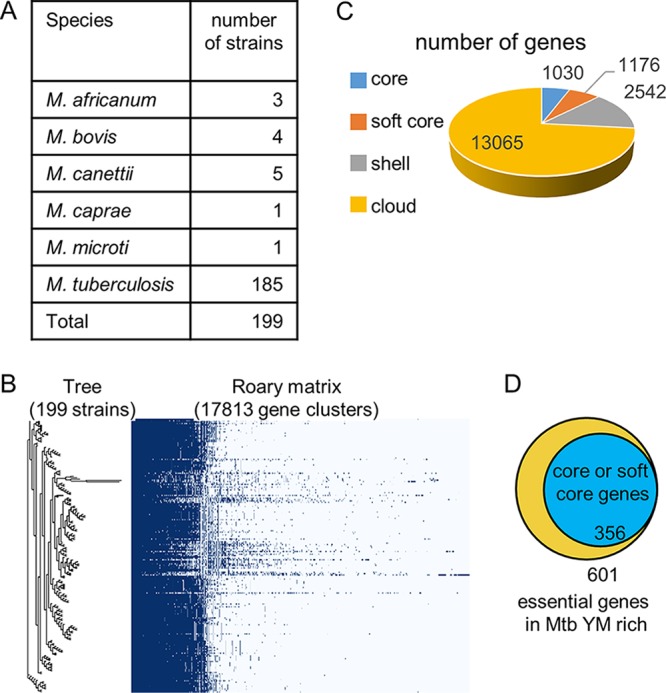
Identification of highly conserved essential genes among virulent M. tuberculosis complex strains. (A) Strains used for comparative genomic analysis. (B) Results for pan-genome analysis by Roary. (C) Number of highly conserved genes among virulent M. tuberculosis complex. Core genes are conserved in >99% of strains, soft core genes are conserved in 95 to 99% of strains, shell genes are conserved in 15 to 95% of strains, and cloud genes are conserved in 0 to 15% of strains. (D) Numbers of genes that are essential for M. tuberculosis H37Rv survival in MtbYM rich medium and highly conserved among virulent M. tuberculosis complex strains.

Essential genes that were identified by Tn-seq were further interrogated through comparative genomic analysis in order to see whether essentiality correlated with high levels of sequence conservation within the M. tuberculosis complex. A total of 226 complete genome sequences of the M. tuberculosis complex were available from the PATRIC database (37). We excluded nonpathogenic strains (e.g., M. tuberculosis H37Ra and Mycobacterium bovis BCG) and used 199 genome sequences for comparative genomic analysis (Fig. 3A; Table S6). We identified a total of 17,813 genes from 199 strains of the M. tuberculosis complex (Fig. 3B and C). Among these genes, 2,206 genes (1,030 core genes and 1,176 soft core genes) were highly conserved in most of the strains (≥95% of strains).

10.1128/mSystems.00070-19.7 TABLE S6 Complete and draft genome sequences of virulent M. tuberculosis complex strains used for comparative genomic analysis. Download Table S6, XLSX file, 0.08 MB. Copyright © 2019 Minato et al. 2019 Minato et al. This content is distributed under the terms of the Creative Commons Attribution 4.0 International license.

Our Tn-seq analysis identified 601 genes as essential in MtbYM rich medium (Data Set S1; Table S2 and smart table [https://biocyc.org/group?id=biocyc14-7907-3764257976]). Among them, we confirmed that at least 60% of the essential genes (356 genes) were included in the list of core/softcore genes (Fig. 3D) (https://biocyc.org/group?id=biocyc14-7907-3764449513). Of note, not all of the target genes for existing antitubercular drugs were categorized as core/soft core genes (Table 1).

### Prediction of essential M. tuberculosis genes *in silico*.

Genome-scale metabolic models have been used to computationally simulate a range of cellular functions (38). We utilized a genome-scale model of M. tuberculosis, iSM810, to predict gene essentiality *in silico* using flux balance analysis (FBA) (39). The principles for FBA have been described previously (40). Briefly, a metabolic network is represented by a stoichiometric matrix that contains reactions and metabolites. A biomass reaction is defined based on experimentally determined amounts of specific metabolites required for cellular growth. The stoichiometric matrix can be converted into a system of linear equations. FBA then solves this system by optimization, typically of the biomass reaction. Constraints on reaction fluxes can be used to simulate metabolite availability or enzyme presence. For example, a reaction’s uptake flux is constrained to zero when the simulated environment does not contain the associated metabolite. Reaction fluxes that simulate metabolite uptake within iSM810 were used to define *in silico* growth media. Uptake was not bounded for metabolites present in the selected growth medium; however, not all medium components were represented within the iSM810 transport reactions (Table S7). Genes were assessed for essentiality by systematically closing the flux on each reaction within iSM810 and assessing simulated biomass production on each medium using FBA. Genes were defined essential if biomass production after knockout was <1e–10.

10.1128/mSystems.00070-19.8 TABLE S7 *In silico* growth predictions of single-gene-knockout mutant strains of M. tuberculosis. Download Table S7, XLSX file, 0.07 MB. Copyright © 2019 Minato et al. 2019 Minato et al. This content is distributed under the terms of the Creative Commons Attribution 4.0 International license.

**FIG 4:**
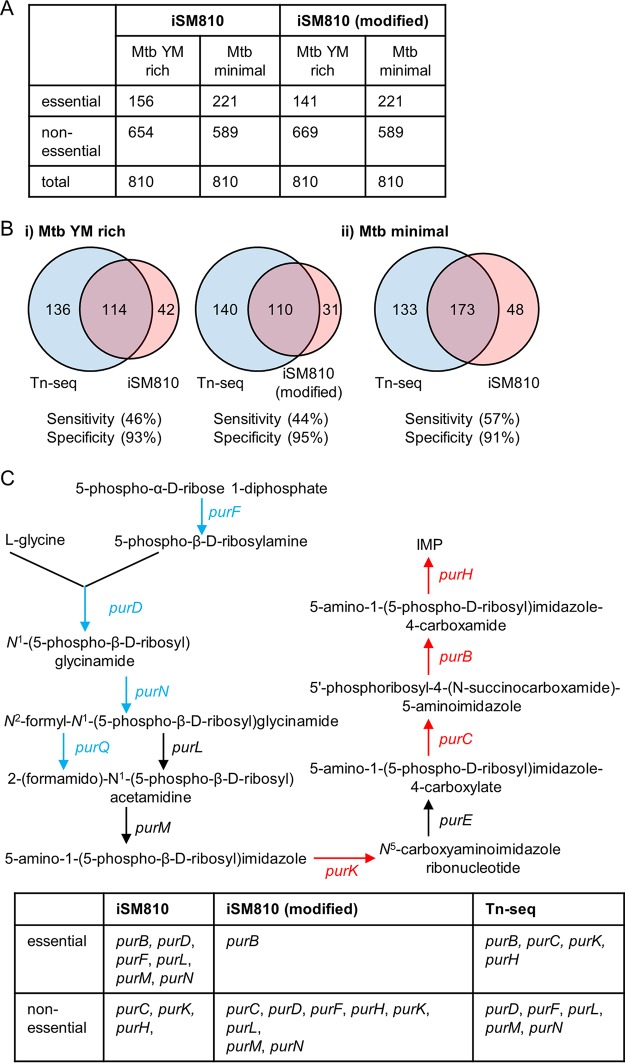
Comparison of Tn-seq-identified essential genes and *in silico*-predicted essential genes. (A) Results for *in silico* gene essentiality prediction by iSM810 and iSM810 (modified). (B) Comparison of Tn-seq-identified essential genes (blue) and iSM810-predicted essential genes (red). (C) Comparison of Tn-seq-identified essential genes and iSM810-predicted essential genes in the purine biosynthesis pathway. Genes in red are essential in both MtbYM rich and Mtbminimal media, genes in blue are essential in Mtbminimal medium but not essential in MtbYM rich medium, and genes in black are nonessential. Information on genes in each pathway was obtained from the BioCyc database (18).

iSM810 includes 810 metabolic genes (including 1 orphan gene) and 938 metabolic reactions (39). Among the 810 genes in iSM810, our FBA analysis predicted that 159 genes were essential in MtbYM rich medium and 221 genes were essential in Mtbminimal medium (Fig. 4A; Table S7). We then compared the genes predicted to be essential by FBA with the genes identified as essential by Tn-seq (Fig. 4B). We found that the sensitivity of the FBA-based gene essentiality prediction was low, as there were a number of genes that were predicted to be essential by FBA but not identified as essential by Tn-seq (Fig. 4B) (https://biocyc.org/group?id=biocyc14-7907-3764439908). For instance, we found that genes related to riboflavin biosynthesis were nonessential in MtbYM rich medium by Tn-seq analysis but were essential *in silico*. We examined why iSM810 could not accurately predict the essentiality of genes in the riboflavin biosynthesis pathway and found that the model lacked a transport reaction for riboflavin (Table S7). Similarly, we found that the model lacked transport reactions for vitamin B_12_, *para*-aminobenzoic acid (PABA), H_2_O, and *myo*-inositol. To investigate whether the addition of these transport reactions could improve gene essentiality prediction by iSM810, we added these transport reactions to the model. These changes fixed multiple mismatches between iSM810 and Tn-seq by causing the model to determine that genes related to thiamine biosynthesis and riboflavin biosynthesis were nonessential in MtbYM rich medium (Fig. 4A and B; Table S7). Allowing PABA uptake caused no changes. After adding transport reactions, the growth rate prediction in MtbYM rich medium increased from 0.055 g/liter/day to 0.0876 g/liter/day.

Unlike the Tn-seq results, the FBA predicted that all genes essential in MtbYM rich medium were also essential in Mtbminimal and failed to predict any genes, such as *metH*, that were essential only in MtbYM rich medium (Table S7). This is expected given the lack of gene regulation implicit in our FBA modeling.

Comparing the sets of essential genes experimentally identified by Tn-seq to those computationally predicted *in silico* highlighted the limitations of current M. tuberculosis metabolic models. For example, we noticed that genes located downstream of the purine biosynthesis pathway were identified as essential by Tn-seq in both MtbYM rich and Mtbminimal media (Fig. 4C). However, there were mismatches between iSM810 and Tn-seq. Among these genes, *purC* is the only gene that was characterized by analysis of targeted gene deletion in M. tuberculosis (41). The *purC* gene was difficult to delete (41), and the *purC* deletion mutant strain of M. tuberculosis showed a notable growth defect compared to the growth of the parent strain even in the presence of hypoxanthine (22). This observation may explain why the *purC* gene was identified as essential by Tn-seq in MtbYM rich medium. However, the other genes in the purine biosynthesis pathway have not been characterized by targeted gene deletion studies. Thus, future studies are necessary to investigate the essentiality of *purK*, *purB*, and *purH* by targeted gene deletion.

We also identified a potential avenue for improvement of the genome-scale model of M. tuberculosis by comparing Tn-seq-identified essential genes and to those predicted *in silico.* We found that many genes in known essential metabolic pathways were not predicted to be essential (e.g., glycolysis, folate metabolism, mycolate biosynthesis, ATP biosynthesis, and several amino acid biosynthesis pathways) (https://biocyc.org/group?id=biocyc13-7907-3766253313). Examination of the biomass function in iSM810 revealed that several known essential metabolites are not connected to the biomass function in iSM810. For example, folate is not connected to the biomass function, and this explains why addition of a PABA transport reaction did not affect any gene essentiality predictions. None of ATP synthase genes in iSM810 (*atpA*, *atpB*, *atpD*, *atpE*, *atpG*, and *atpH*) were predicted as essential because they were linked to the same reaction using “or” Boolean logic, meaning that the presence of one gene was sufficient to allow the reaction to proceed. Thus, further improvement of iSM810 for more accurate prediction of essential genes may be achieved by connecting such essential metabolites to the biomass function.

### Conclusions.

In this study, we utilized functional genomics and comparative genomics approaches to identify essential M. tuberculosis genes. We identified distinct sets of essential and conditionally essential genes by different approaches and with different growth conditions. In addition, comparison of essential genes identified by the functional genomics approach to *in silico*-predicted essential genes highlighted current gaps in our knowledge regarding M. tuberculosis metabolism. Our study provides a promising platform to shed new light on essential cellular functions in M. tuberculosis that can lead to the discovery of novel targets for antitubercular drugs.

## MATERIALS AND METHODS

### Media and growth conditions.

M. tuberculosis H37Rv was grown aerobically at 37°C in Middlebrook 7H9 medium supplemented with oleate-albumin-dextrose-catalase (OADC; 10%, vol/vol), glycerol (0.2%, vol/vol), and tyloxapol (0.05%, vol/vol) unless otherwise noted. Mtbminimal medium (0.2%, vol/vol, glycerol as a sole carbon source) (21) and MtbYM rich medium (see Table S1 in the supplemental material) agar plates were used to generate M. tuberculosis H37Rv transposon libraries. Tyloxapol (0.05%, vol/vol) was added to the both Mtbminimal and MtbYM rich agar plates.

### Construction of saturated transposon libraries of M. tuberculosis.

Transposon mutagenesis was performed as previously described (42). Mycobacteriophage phAE180 (42) was used to transduce a mariner derivative transposon, Tn*5371* (43), into M. tuberculosis H37Rv that had been grown until the mid-log growth phase (optical density at 600 nm [OD_600_], 0.5). Transduced M. tuberculosis H37Rv was spread on an Mtbminimal medium plate and MtbYM rich medium plate and incubated at 37°C for 2 to 3 weeks. To generate a transposon library in saturated size, at least 150,000 colonies were collected from each plate. Each transposon library was aliquoted and stored at −80°C. Each transposon library was generated in duplicate.

### Tn-seq.

Genomic DNA (gDNA) was prepared from each sample as previously described (44). gDNA was then fragmented using an S220 acoustic DNA shearing device (Covaris). After the shearing, adapters were added using an Illumina TruSeq Nano DNA library prep kit according to the manufacturer’s instructions. Transposon junctions were amplified by using a transposon-specific primer, Mariner_1R_TnSeq_noMm (TCGTCGGCAGCGTCAGATGTGTATAAGAGACAGCCGGGGACTTATCAGCCAACC [the transposon is underlined]), and a p7 primer (CAAGCAGAAGACGGCATACGAG) with a HotStarTaq master mix kit (Qiagen) and the following PCR conditions (94°C for 3 min, 30 cycles of 94°C for 30 s, 65°C for 30 s, and 72°C for 60 s, and 72°C for 10 min). The transposon junction-enriched sample was diluted 1:50 with water and then amplified to add the flow cell adapter and i5 index to the enriched transposon-containing fragments using the following primers: an i5 indexing primer, AATGATACGGCGACCACCGAGATCTACACXXXXXXXXTCGTCGGCAGCGTC (where XXXXXXXX denotes the position of the 8-bp index sequence), and a p7 primer, CAAGCAGAAGACGGCATACGAG.

The amplification reaction mixture was as follows: 5 μl template DNA (from PCR 1), 1 μl nuclease-free water, 2 μl 5× KAPA HiFi buffer (Kapa Biosystems), 0.3 μl 10 mM deoxynucleoside triphosphates (dNTPs) (Kapa Biosystems), 0.5 μl dimethyl sulfoxide (DMSO) (Fisher Scientific), 0.2 μl KAPA HiFi polymerase (Kapa Biosystems), 0.5 μl i5 indexing primer (10 μM), and 0.5 μl p7 primer (10 μM). Cycling conditions were as follows: 95°C for 5 min, followed by 10 cycles of 98°C for 20 s, 63°C for 15 s, and 72°C for 1 min, followed by a final extension at 72°C for 10 min.

Amplification products were purified with AMPure XP beads (Beckman Coulter), and the uniquely indexed libraries were quantified using a Quant-IT PicoGreen double-stranded DNA (dsDNA) assay (ThermoFisher Scientific). The resulting fragment size distribution was assessed using a Bioanalyzer (Agilent Technologies). The resultant Tn-seq library was sequenced using a HiSeq 2500 high-output (HO), 125-bp paired-end (PE) run using v4 chemistry (Illumina).

### Tn-seq analysis.

Sequence reads were trimmed using CutAdapt (45). We first trimmed sequence reads for transposon sequences (CCGGGGACTTATCAGCCAACCTGT) at the 5′ ends. Reads that did not contain a transposon sequence at the 5′ end were discarded. After the 5′-end-trimming process, all the sequence reads began with TA. We then trimmed sequence reads for adaptor sequences ligated to the 3′ end (GATCCCACTAGTGTCGACACCAGTCTC). After the trimming, we discarded the sequence reads that were shorter than 18 bp. The default error rate of 0.1 was used for all for all trimming processes.

The trimmed sequence reads were mapped (allowing a 1-bp mismatch) to the M. tuberculosis H37Rv genome (GenBank accession number AL123456.3) using Bowtie 2 (46). The number of reads at each TA site was counted and converted to the .wig format, the input file format for TRANSIT (14), using a custom Python script (Text S1). Subsequent statistical analysis for gene essentiality (Bayesian/Gumbel method, HMM, and resampling method) were performed using TRANSIT (version, 2.0.2) (14).

10.1128/mSystems.00070-19.1 TEXT S1 Custom Python script used in this analysis. Download Text S1, TXT file, 0 MB. Copyright © 2019 Minato et al. 2019 Minato et al. This content is distributed under the terms of the Creative Commons Attribution 4.0 International license.

The Bayesian/Gumbel method determines posterior probability of the essentiality of each gene (shown in the zbar column in Data Set S1). When the value is 1 or near 1 within the threshold, the gene is called essential. When the value is 0 or near 0, the threshold, gene is called nonessential. When the value is between the two thresholds, neither near 0 nor 1), the gene is called uncertain. When the value is −1, the gene is called small because the gene is considered too small to determine posterior probability of essentiality. Thus, we analyzed the essentialities of small and uncertain genes by HMM. All essential genes identified from uncertain or small genes are listed in Table S2.

A total of 601 genes (https://biocyc.org/group?id=biocyc14-7907-3764257976) essential for M. tuberculosis survival in the MtbYM rich medium (542 genes by the Bayesian/Gumbel method and an additional 59 genes by the HMM) were used for comparisons to the results of two past Tn-seq studies (8, 9). Lists of essential genes identified by past studies were obtained from Data Set S1 in reference 8 and from reference 9.

### FBA.

Flux balance analysis (FBA) solutions were obtained using a simulated environment designed to mimic the MtbYM rich medium designed for this study. This was done by altering uptake boundaries to match the concentration of each metabolite. Most metabolites present in the media were given unlimited boundaries, because these metabolites were not expected to be limiting and also because they were present at an undefined concentration in the MtbYM rich medium, due to their source being Casamino Acids. Metabolites added to the MtbYM rich medium in known concentrations were bounded in FBA at those concentrations.

The iSM810 model contains 938 metabolic reactions and 810 genes (including 1 orphan gene) (39). The biomass reaction originally described for iSM810 was chosen to define growth. FBA was performed using the COBRA Toolbox Matlab package (47, 48). The unconstrained uptake fluxes were set to 1. Gene essentiality was assessed using the COBRA Toolbox single-gene-deletion function in Matlab. Through single-gene deletion, reactions associated with each gene were systematically closed and the model was optimized for biomass production. Any biomass accumulation of >1e–10 (which could occur due to numerical errors) was defined as growth, and the gene was classified as nonessential. A biomass accumulation of <1e–10 resulted in a gene being called essential. All FBA optimizations were done using the Gurobi Optimizer 7.0 software under a free academic license (Gurobi Optimization, Inc.).

### Comparative genomic analysis.

The following comparative genomic analysis was carried out as previously reported (49). In brief, the pan- and core genomes were defined using Roary software (50). Complete and draft genome sequences of pathogenic strains (nonhighlighted strains were used and obtained from the PATRIC database [accessed 1 October 2017]) summarized in Table S6 were reannotated to generate gff3 files using PROKKA version 1.1.12 software (51) and to include annotation of a reference strain, H37Rv. Homologous proteins (i.e., protein families) were clustered using the CD-Hit and MCL algorithms. The BLASTp cutoff value was set at 95%. The numbers of core and pan-genome protein families were estimated via genome sampling up to the number of input genomes at the default setting in Roary (Data Set S2).

10.1128/mSystems.00070-19.10 DATA SET S2 Presence or absence of genes within complete genomes of the Mycobacterium tuberculosis complex analyzed by Roary. Download Data Set S2, XLSX file, 10.8 MB. Copyright © 2019 Minato et al. 2019 Minato et al. This content is distributed under the terms of the Creative Commons Attribution 4.0 International license.

### Data availability.

Raw sequencing data in FASTA format is publicly available for download through the Data Repository for the University of Minnesota at http://hdl.handle.net/11299/203632.
